# A Patient With Hemoglobin SC Disease and Acute Ischemic Stroke Presenting With Altered Mental Status

**DOI:** 10.7759/cureus.18610

**Published:** 2021-10-08

**Authors:** Aye M Thida, Kitson Deane, Mateus Fernandes, Htun M Aung, Pouyan Gohari

**Affiliations:** 1 Department of Medicine, Woodhull Medical and Mental Health Center, Brooklyn, USA; 2 Department of Medicine, Interfaith Medical Center, Brooklyn, USA; 3 Department of Hematology and Oncology, Woodhull Medical and Mental Health Center, Brooklyn, USA

**Keywords:** acute neurological deficit, red cell exchange transfusion, altered mental status evaluation, acute ischemic stroke, hemoglobin sc disease

## Abstract

A 57-year-old woman with a history of hypertension, diabetes mellitus, obesity, asthma, and hemoglobin SC disease presented to the emergency department by her home health aide after she was found having altered mental status. According to her home health aide, the patient was responding with “Ok” to her questions for more than a day. The hemoglobin on admission was 8.5 g/dL. A magnetic resonance imaging (MRI) without contrast of the brain showed acute cortical infarcts superimposed on the old infarct zone. The patient received 1 unit of packed red blood cells and a session of exchange transfusion, in addition to aspirin, clopidogrel, and atorvastatin during the hospital stay. When a patient known to have sickle cell disease presents with acute neurological deficits, the first consideration is usually acute ischemic stroke due to vaso-occlusion in the cerebral vessels. However, it is essential to not overlook other potential causes of acute neurological deficits.

## Introduction

Hemoglobin SC (HbSC) disease is due to co-inheritance of the hemoglobin S (HbS) and hemoglobin C (HbC) beta-globin gene mutations [1]. It accounts for nearly 30% of sickle cell disease (SCD) in the United States of America and the United Kingdom [2,3]. On the other hand, it accounts for more than 50% of SCD in some areas of West Africa where HbC originated [4]. Because many people with HbSC disease emigrate from countries without newborn screening, they may only be diagnosed when a disease-related complication arises.

Although HbSC disease is considered a milder variant of SCD, it is associated with multiple systemic complications such as painful crises, ocular, splenic, neurological, cardiovascular, and pulmonary complications, and priapism [5]. Moreover, the Cooperative Study of Sickle Cell Disease (CSSCD) in 2002 revealed that the prevalence of overt and silent stroke in patients with HbSC disease was 0% and 5.8%, respectively [6]. However, recent retrospective studies in 2015 and 2019 noted a higher prevalence of neurovascular complications in these patients, with an overall prevalence of 17.7% (2.9% overt stroke, 8.8 to 13.5% silent stroke, and 5.9% vasculopathy) [7,8].

We describe the case of a 57-year-old woman with HbSC disease presenting with altered mental status, which turned out to be due to acute ischemic stroke.

## Case presentation

A 57-year-old woman with a history of hypertension, diabetes mellitus, obesity, asthma, and HbSC disease presented to the emergency department after she was found having altered mental status. According to her home health aide, when she visited the patient a day before, the patient responded with “Ok” to pleasantries. The following day, she found that the patient continued to respond with “Ok” to her questions. She stated that at baseline, the patient was able to communicate well, ambulate with a cane, and required assistance with activities of daily living. The patient also had a history of smoking a pack of cigarettes per day for about 20 years.

On triage, the patient had a body temperature of 98.2 degrees Fahrenheit, blood pressure of 143/96 mmHg, heart rate of 113 beats per minute, respiratory rate of 18 breaths per minute, oxygen saturation of 96% in room air, and blood sugar of 213 mg/dL. The patient was not articulating or able to communicate history. Physical examination did not reveal significant findings other than altered mental status. There were no cranial nerve deficits.

Investigations

Initial laboratory investigations revealed mild leukocytosis, normochromic normocytic anemia, elevated reticulocyte count, total bilirubin, lactate dehydrogenase, and possibly acute kidney injury (Table 1). Urine toxicology was negative. An electrocardiogram showed sinus tachycardia. There was no radiographic evidence of acute pulmonary disease on the chest X-ray. A computed tomography (CT) scan without contrast of the head was performed in the emergency department, which did not reveal mass effect, midline shift, or intracranial hemorrhage.

**Table 1:** Initial laboratory investigations H, high; L, low

Investigations	Results	Normal range
White blood cells	12.36 (H)	4.5–11x10^3^/µL
Red blood cells	2.85 (L)	4.0–5.7x10^6^/µL
Hemoglobin	8.5 (L)	13.0–17.0 g/dL
Hematocrit	23.2 (L)	39–53 %
Mean corpuscular volume	81.4	80–100 fL
Mean corpuscular hemoglobin	29.8	26–33 pg
Mean corpuscular hemoglobin concentration	36.3 (H)	30.5–36.0 g/dL
Red cell distribution width	18.2 (H)	11.5–15.1%
Reticulocyte %	5.08 (H)	0.50–2.00%
Absolute reticulocyte count	0.14 (H)	0.05–0.10x10^6^/µL
Platelet count	184	130–400x10^3^/µL
Sodium	143	136–145 mmol/L
Potassium	4	3.5–5.1 mmol/L
Chloride	103	98.0–107.0 mmol/L
Carbon dioxide	24	223–31 mEq/L
Blood urea nitrogen	27 (H)	8.4–25.7 mg/dL
Creatinine	1.08	0.72–1.25 mg/dL
Glucose	213	80–115 mg/dL
Calcium	10.8 (H)	8.8–10.0 mg/dL
Total bilirubin	2.1 (H)	0.2–1.2 mg/dL
Direct bilirubin	0.7 (H)	0.0–0.5 mg/dL
Aspartate transaminase	23	5–34 U/L
Alanine transaminase	18	10–55 U/L
Alkaline phosphatase	97	40–150 U/L
Lactate dehydrogenase	517 (H)	80–200 U/L
Hemoglobin A	0.0 (L)	95.8–98.0%
Hemoglobin A2	3.7 (H)	2.0–3.2%
Hemoglobin C	43.4 (H)	≤0.0%
Hemoglobin S	51.3 (H)	≤0.0%
Hemoglobin variant 1	0.6 (H)	≤0.0%

Treatment

As the patient had multiple risk factors for stroke, she was admitted to the medical telemetry unit for a suspected cerebrovascular accident and was given aspirin 325 mg and atorvastatin 80 mg. The patient was deemed not a candidate for thrombolytic therapy, given that the duration of acute stroke was over 4.5 hours.

**Figure 1:**
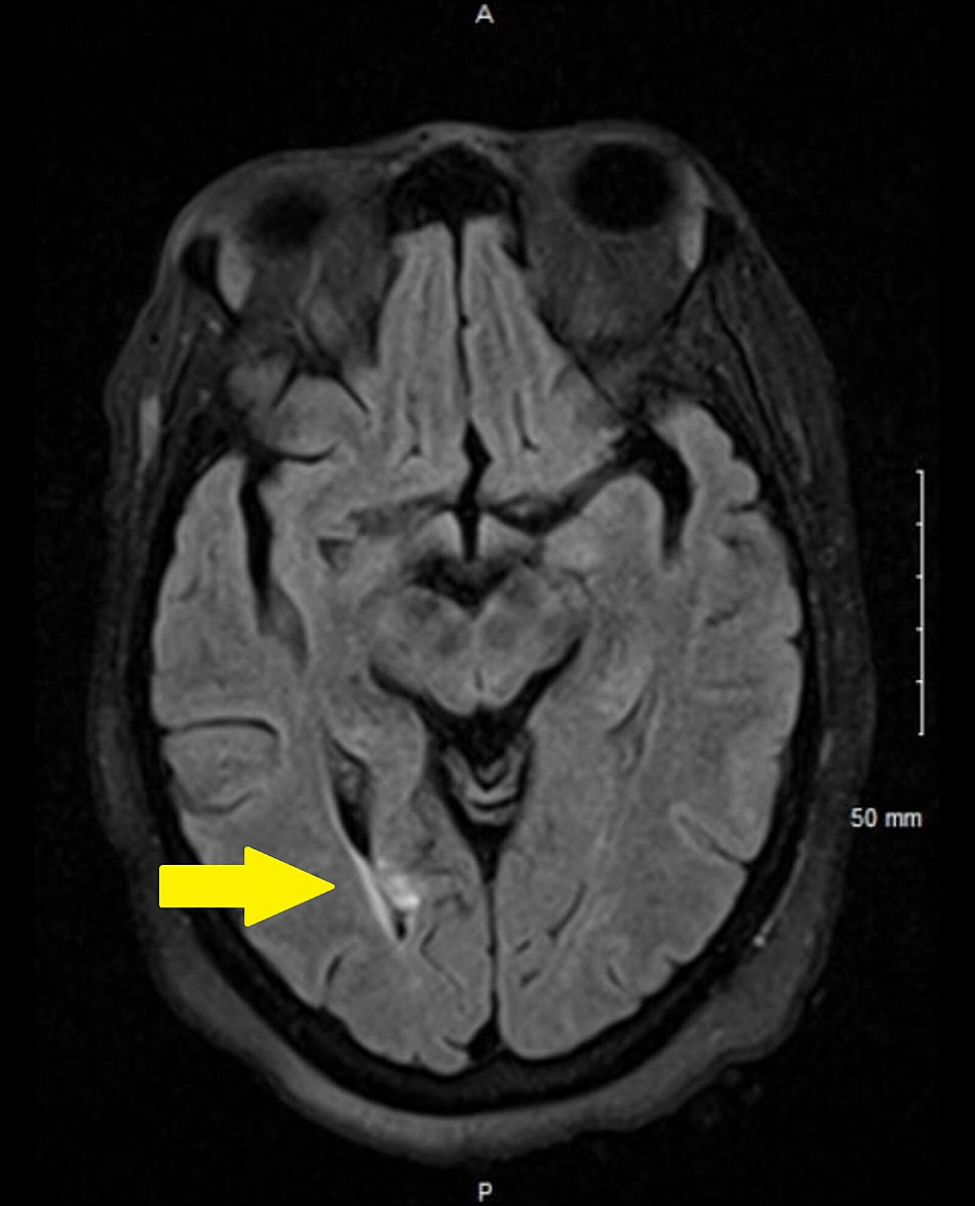
MRI without contrast of the brain (T2-FLAIR) showed acute cortical infarct which appeared as an area of increased brightness (hyperintense) in the right parieto-occipital region. T2-FLAIR, T2-weighted fluid-attenuated inversion recovery

On day 2, the patient was alert, oriented, and able to answer questions appropriately but kept forgetting certain things. She claimed that she lived alone, had a stroke in the past, and was ambulating with a cane. On physical examination, there was a left-sided weakness of 4 out of 5, but no other abnormalities. A magnetic resonance imaging (MRI) without contrast of the brain (T2 FLAIR [T2-weighted fluid-attenuated inversion recovery]) showed a small old medial right parieto-occipital infarct with tiny acute cortical infarcts superimposed on the old infarct zone, and a small old right callosal and adjacent right cingulate gyrus focal infarct (Figure 1). Clopidogrel 75 mg was started. A cerebral CT angiogram was within normal limits. A carotid duplex ultrasound revealed less than 50% stenosis of the right internal carotid artery and a normal left internal carotid artery. An echocardiogram showed an ejection fraction of 65% and right ventricular systolic pressure of 88 mmHg with negative bubble study.

The hematology team was consulted on day 3. As the patient was presenting with acute ischemic stroke in the setting of HbSC disease and hemoglobin on admission was 8.5 g/dL (was between 9 to 10 g/dL a year ago), she received 1 unit of packed red blood cells (RBCs). On day 4, she underwent a session of exchange transfusion with 10 units of packed RBCs without any complications. Hemoglobin electrophoresis following exchange transfusion revealed 14.4% HbS and 12.2% HbC from a baseline of 51.3% HbS and 43.4% HbC on the day of admission.

Outcome and follow-up

She remained alert, oriented, and ambulating with a cane. No neurological deficit was noted. She was discharged to her home and provided a follow-up appointment in the clinic. Two months after discharge, the patient presented to the hematology clinic with chronic right shoulder pain, but there was no focal neurological deficit. A repeat blood test revealed stable results, including hemoglobin of 9.6 mg/dL, reticulocyte % of 2.95%, and lactate dehydrogenase of 188 U/L.

## Discussion

The pathophysiology of HbSC disease is believed to be modulated by interactions between HbS and HbC as well as RBC dehydration from altered membrane transporter function [5]. It is important to note that HbS concentration increases when RBCs are dehydrated [9]. At moderate-to-high level, HbS subunits polymerize and form long, rigid molecules, which persist as RBCs enter the microcirculation and obstruct the flow causing complications [10]. Moreover, the lifespan of RBCs in HbSC disease is twice as long as that of hemoglobin SS (HbSS) disease (29 versus 15 days) [11]. These patients have a higher mean hemoglobin level and a lower absolute reticulocyte count than those with HbSS disease. More than 70% of people with HbSC disease have anemia, but only 10% have hemoglobin lower than 10 g/dL. Irreversibly sickled cells are rare, and blood viscosity is higher than that in HbSS disease [10].

The prevalence of neurovascular complications in patients with HbSC disease is believed to be higher than previously reported [7,8]. Patients with underlying hypertension (odds ratio [OR]: 4.1; 95% confidence interval [CI]: 2.9-5.7; p ≤ 0.0001), hyperlipidemia (OR: 6.9; 95% CI: 2.9-14; p ≤ 0.0001), renal disease (OR: 4.2; 95% CI: 2.4-6.8; p ≤ 0.0001), and atrial fibrillation (OR: 4.9; 95% CI: 2.2-9.5; p ≤ 0.0005) are at increased risk for ischemic stroke [12]. On the other hand, hemorrhagic stroke is more common in those with underlying hypertension (OR: 7.7; 95% CI: 4.7-12.7; p ≤ 0.0001), renal disease (OR: 7.2; 95% CI: 3.4-13.9; p ≤ 0.0001), and coagulopathy (OR: 9.1; 95% CI: 2.8-22.4; p ≤ 0.0005).

Furthermore, the rate of stroke is much higher in middle-aged and elderly adults than in children and young adults [12]. Strouse et al. reported that the rate of stroke is 310/100,000 person-years in children less than 18 years of age, 360/100,000 person-years in young adults aged 18 to 34 years, 1,160/100,000 person-years in middle-aged adults aged 35 to 64 years, and 4,700/100,000 person-years in elderly adults more than 64 years of age. Although annual transcranial Doppler (TCD) screening and intervention with chronic RBC transfusions have reduced ischemic stroke rates in children with HbSS disease, there is no evidence that children with HbSC disease should undergo TCD screening for primary stroke prevention [13].

When a patient known to have SCD presents with acute neurological deficits, the first consideration is usually acute ischemic stroke due to vaso-occlusion in the cerebral vessels. However, it is essential not to overlook other potential causes of acute neurological deficits such as acute hemorrhagic stroke, acute meningitis, brain abscess, seizure, migraine, and cerebral venous sinus thrombosis. A rapid initial clinical assessment should be performed followed by neuroimaging to differentiate ischemic stroke from hemorrhagic stroke and to exclude other differential diagnoses.

The American Society of Hematology 2020 guideline panel recommends exchange transfusion versus simple transfusion for children or adults with SCD and acute neurological deficits including transient ischemic attack [14]. Simple transfusion can be performed if exchange transfusion is not feasible within 2 hours of presentation for medical care and hemoglobin level is ≤8.5 g/dL to avoid delays in treatment while planning for manual exchange transfusion or automated apheresis. Besides, the consideration of intravenous tissue plasminogen activator (tPA) should not delay prompt simple or exchange blood transfusion therapy.

While simple transfusion raises hemoglobin and blood viscosity, exchange transfusion removes sickled RBCs and replaces them with allogeneic RBCs, thus lowering or minimally increasing blood viscosity [5]. Therapeutic targets for RBC transfusion in HbSC disease are derived from HbSS disease, where the goals are hemoglobin of 10 g/dL and HbS of <30%. This is because higher baseline hemoglobin in HbSC disease may increase blood viscosity and hemoglobin after simple transfusion while reducing the HbS% marginally.

For the prevention of complications, there is low-level evidence of safety and efficacy on the use of disease-modifying RBC transfusions, phlebotomy, and hydroxycarbamide [15-17].

## Conclusions

We described the management of a patient with HbSC disease who presented with altered mental status. After she was diagnosed with acute ischemic stroke, she received 1 unit of packed RBCs and a session of exchange transfusion, in addition to aspirin, clopidogrel, and atorvastatin during the hospital stay. When a patient known to have SCD presents with acute neurological deficits, the first consideration is usually acute ischemic stroke due to vaso-occlusion in the cerebral vessels. However, it is essential not to overlook other potential causes of acute neurological deficits.
